# *Strongyloides stercoralis* Hyperinfection Presenting as Diffuse Alveolar Hemorrhage in an Endemic Region: A Case Report

**DOI:** 10.3390/tropicalmed11050133

**Published:** 2026-05-14

**Authors:** Juan Camilo Motta, Manuel Alejandro Delgado, Jacqueline Mugnier-Quijano

**Affiliations:** 1 Internal Medicine Service, Fundación Cardioinfantil—Instituto de Cardiología, Bogotá 110131, Colombia; 2 Infectious Disease Service, Universidad Nacional de Colombia, Bogotá 111321, Colombia; 3 School of Medicine and Health Sciences, Universidad del Rosario, Bogotá 111321, Colombia; manuel.delgado@urosario.edu.co; 4 Department of Pathology, Fundación Cardioinfantil—Instituto de Cardiología, Bogotá 110131, Colombia; jmugnier@lacardio.org

**Keywords:** *Strongyloides stercoralis*, hyperinfection syndrome, diffuse alveolar hemorrhage

## Abstract

Background: *Strongyloides stercoralis* is a soil-transmitted helminth capable of establishing chronic infection through an autoinfective cycle, with the potential to progress to life-threatening hyperinfection, particularly in immunocompromised individuals. Case Presentation: We report the case of a 70-year-old man from an endemic region in Colombia with metastatic urothelial carcinoma who developed hyperinfection syndrome following corticosteroid therapy for spinal cord compression. The patient presented with progressive respiratory failure and diffuse alveolar hemorrhage. Chest imaging showed bilateral ground glass opacities, and bronchoalveolar lavage revealed numerous larvae consistent with *S. stercoralis*, confirming the diagnosis. Despite supportive care and broad-spectrum antimicrobial therapy, the patient experienced rapid clinical deterioration and died. Conclusions: This case highlights the importance of considering strongyloidiasis in the differential diagnosis of diffuse alveolar hemorrhage in endemic settings, particularly in patients receiving corticosteroids. Early recognition and timely treatment are essential to reduce the high associated mortality. Preventive strategies, including targeted screening or empiric ivermectin administration prior to immunosuppression, should be considered in high-risk populations.

## 1. Introduction

*Strongyloides stercoralis* is a soil-transmitted intestinal nematode with a worldwide distribution, primarily affecting tropical regions. Its estimated global prevalence is 8.1%, representing approximately 613.9 million individuals [1]. Studies conducted in Latin America have reported high prevalence rates (>20%) in countries such as Argentina, Ecuador, Venezuela, Peru, and Brazil [1,2]. However, these estimates are highly heterogeneous, and epidemiological data remain limited or scarce in other countries, including Colombia, Mexico, and Bolivia [2]. This underscores a substantial gap in the regional epidemiological landscape.

Infection occurs when filariform larvae penetrate the skin, migrate hematogenously to the lungs, ascend the respiratory tract, and are subsequently swallowed into the small intestine, where they mature and reproduce [3]. A subset of larvae differentiates into autoinfective forms that can perpetuate the life cycle within the host, enabling long-term persistence without exogenous reinfection. This endogenous autoinfection accounts for the chronic nature of the disease and increases the risk of hyperinfection, particularly in immunocompromised individuals [3,4].

The transition between larval stages is regulated by conserved signaling pathways in nematodes, including cyclic guanosine monophosphate (cGMP), insulin-like signaling (IIS), and transforming growth factor beta (TGF-β), as well as nuclear receptors such as DAF-12 that respond to steroid-derived ligands [5,6]. The clinical relevance in this case lies in the interaction between exogenous corticosteroids and parasite development, which likely contributed to rapid disease progression.

Corticosteroid exposure is a central factor in the development of hyperinfection. In this patient, dexamethasone likely contributed to disease progression through two complementary mechanisms. First, corticosteroids impair protective host immunity, particularly T cell-mediated and Th2 responses, including interleukin-5 production and eosinophil activation, which are essential for helminth control [7]. Second, glucocorticoid-derived metabolites may mimic ecdysteroid-like signals, modulate parasite developmental pathways, and promote the transformation of rhabditiform larvae into autoinfective filariform larvae within the host [7,8].

Most chronic infections remain asymptomatic or are associated with nonspecific gastrointestinal and cutaneous manifestations. However, in the presence of risk factors such as immunosuppression, chemotherapy, or corticosteroid exposure, the autoinfective cycle can be exacerbated, resulting in hyperinfection syndrome or disseminated strongyloidiasis [9,10]. These manifestations carry a high mortality risk, especially when complicated by alveolar hemorrhage, an uncommon but life-threatening condition [11,12].

In endemic settings such as Colombia, where transmission-favoring socioeconomic conditions persist—including poor sanitation, limited access to safe water, rural environmental exposure to contaminated soil, overcrowding, and restricted access to healthcare—strongyloidiasis remains underdiagnosed and underestimated. Accordingly, we report this case to emphasize the importance of considering this entity in the differential diagnosis of diffuse alveolar hemorrhage, particularly in patients with recent corticosteroid exposure or other forms of immunosuppression.

## 2. Case Presentation

A 70-year-old man from San Sebastián, Magdalena (a rural area in northern Colombia, 23 m above sea level, tropical climate), presented with a two-month history of lower-limb pain. He worked as a construction laborer and had a history of chronic hypertension, well controlled with oral valsartan 80 mg/day. There was no history of illicit drug use, including crack cocaine, and the patient was not receiving anticoagulant or antiplatelet therapy. The pain was described as lancinating, progressively increasing in intensity, and predominantly affecting the right side, with associated bilateral weakness, more pronounced on the right.

**Figure 1:**
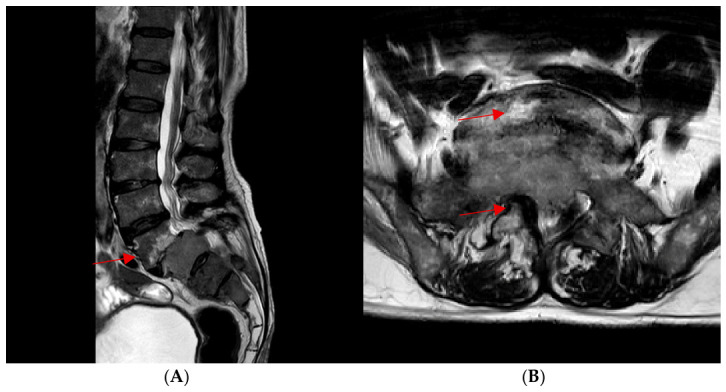
Lumbosacral MRI. (**A**) Sagittal T2-weighted image demonstrating an expansile lesion involving the S1–S3 segments with severe spinal canal stenosis and soft tissue extension. (**B**) Axial T2-weighted image confirming canal compromise and mass effect on the cauda equina. Arrows highlight the lesion and neural compression.

On initial physical examination, decreased sensation in the right lower limb and bilateral motor weakness, predominantly on the right, were documented. HIV serology was negative. Magnetic resonance imaging of the lumbosacral spine revealed an expansile mass with neoplastic features involving the S1–S3 segments. The lesion caused severe spinal canal stenosis, extended into surrounding soft tissues, and encased the ipsilateral right cauda equina nerve roots (Figure 1). These findings were consistent with an oncologic emergency due to spinal cord compression syndrome secondary to an expansile lesion.

Based on clinical and radiological findings, treatment with intravenous dexamethasone 8 mg every 12 h was initiated, and a biopsy of the lesion was performed. Histopathological analysis demonstrated a poorly differentiated carcinoma consistent with urothelial origin. The patient was found to have metastatic disease and was considered for palliative radiotherapy and further oncologic management.

On hospital day 25, the patient developed progressive respiratory deterioration with refractory hypoxemia and increasing oxygen requirements. This was accompanied by hemodynamic instability requiring orotracheal intubation and vasopressor support with norepinephrine. Laboratory findings demonstrated leukocytosis, significant anemia, thrombocytopenia, electrolyte disturbances, and mild transaminase elevation (Table 1), consistent with severe systemic involvement.

**Figure 2:**
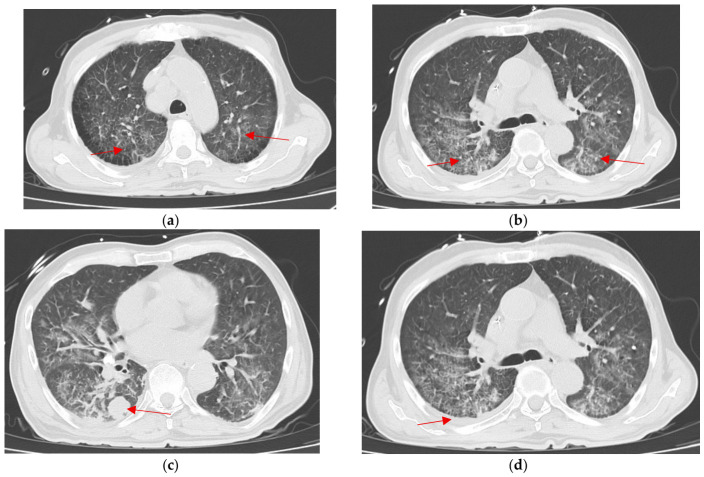
(**a**,**b**) Chest computed tomography demonstrates alveolar consolidation with a peribronchovascular distribution, predominantly involving the dependent regions and affecting both lung parenchyma. These findings are associated with multilobar areas of ground glass opacities, defined as regions of increased lung attenuation with preservation of underlying bronchovascular structures (arrows) (**c**) Additionally, a solid nodular lesion measuring approximately 23 × 20 mm is identified in the posterior segment of the lower right lobe, (**d**) A small right-sided pleural effusion is present, along with subsegmental atelectasis in both lower lobes.

Chest imaging revealed diffuse interstitial reticular opacities with bilateral ground-glass attenuation, predominantly in a central distribution, suggestive of infectious pulmonary involvement (Figure 2). Empirical antimicrobial therapy with meropenem and linezolid was started. Blood cultures were obtained and remained negative. Due to persistent clinical deterioration and suspicion of atypical pathogens, trimethoprim/sulfamethoxazole was added, and bronchoalveolar lavage (BAL) was performed.

**Figure 3:**
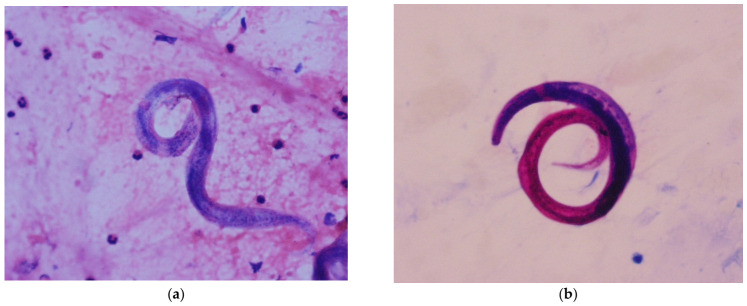
Cytological examination BAL. (**a**) Hematoxylin and eosin (H&E), 10× magnification, demonstrating a filariform (L3) larva consistent with *Strongyloides stercoralis.* (**b**) Periodic acid–Schiff (PAS), 10× magnification, highlighting a filariform (L3) larva consistent with *Strongyloides stercoralis*.

BAL revealed mildly congestive mucosa throughout both bronchial trees, with preserved caliber and normal lobar and segmental anatomy. Bilateral blood clots were observed within the tracheobronchial tree, without evidence of active bleeding, and were aspirated. At the level of the left lower lobe, diffuse alveolar hemorrhage (DAH) was identified. BAL was performed in the lateral basal segment of the left lower lobe (LB9), yielding hemorrhagic fluid. BAL analysis demonstrated a high proportion of hemosiderin-laden macrophages (80%), confirming the presence of DAH. Cytological examination revealed numerous *Strongyloides stercoralis* larvae (Figure 3), establishing the diagnosis of hyperinfection syndrome.

To provide a clear overview of the chronological sequence of clinical events during the patient’s course, a summary timeline is presented in Table 2. Given the presence of DAH, alternative etiologies—including infectious causes, immune-mediated vasculitis, and malignancy-related processes—were considered. The patient received oral ivermectin at a dose of 200 µg/kg once daily for two consecutive doses. However, due to rapid clinical deterioration and early death, a complete course of antiparasitic therapy could not be achieved.

## 3. Discussion

DAH is an uncommon but severe pulmonary manifestation of *Strongyloides stercoralis* hyperinfection. Although pulmonary involvement occurs in a high proportion of severe cases, hemorrhagic complications are rarely reported and are typically associated with advanced disease and poor outcomes [13,14]. The underlying mechanism is thought to involve direct larval migration through the alveolar capillary membrane, resulting in mechanical disruption, inflammatory injury, and bleeding [15,16]. This case highlights the diagnostic value of bronchoscopy and the severity of the disease. Notably, the absence of eosinophilia and negative stool examinations are well-documented features of hyperinfection [17].

A key diagnostic challenge in this case was the absence of eosinophilia, a well-described feature of severe strongyloidiasis [17]. As observed in the previously discussed Colombian cases by Vinueza et al. [18] and Rivera et al. [19], eosinophilia was likewise absent despite confirmed hyperinfection, reinforcing that its absence should not be used to exclude the diagnosis in at-risk patients.

*Strongyloides stercoralis* hyperinfection results from an accelerated autoinfective cycle, leading to increased numbers of filariform larvae (L3) and dissemination beyond the gastrointestinal tract. In this case, corticosteroid exposure likely triggered this process by impairing host immunity and promoting larval maturation [9,10]. In the present case, corticosteroid exposure and malignancy-associated immunosuppression likely acted synergistically, facilitating uncontrolled larval replication and pulmonary involvement.

This pattern is consistent with previously reported cases in Colombia. Vinueza et al. described a fatal case of disseminated strongyloidiasis in which corticosteroid exposure likely precipitated rapid clinical deterioration, culminating in polymicrobial sepsis and multiorgan failure [18]. Similarly, Rivera et al. reported a fatal hyperinfection syndrome in a patient with HTLV-1 infection and ulcerative colitis, where the combination of immune dysregulation and corticosteroid therapy led to rapid dissemination with pulmonary and gastrointestinal involvement [19]. Together with the present case, these reports highlight the critical role of corticosteroid exposure in triggering severe disease in vulnerable hosts within endemic settings.

DAH encompasses a broad differential diagnosis that includes both immune-mediated and non-immune etiologies. Among immune causes, ANCA-associated vasculitis, systemic lupus erythematosus, and anti-glomerular basement membrane disease are the most frequent, typically presenting with pulmonary capillaritis [20]. In contrast, non-immune causes include infections, coagulopathies, drug-induced lung injury, and malignancy-related processes [21].

In this patient, several features argued against an immune-mediated etiology, including the absence of clinical or laboratory evidence of systemic autoimmune disease. Similarly, no history of anticoagulant or antiplatelet therapy was present, and there was no evidence of coagulopathy, making alternative causes less likely.

The identification of *Strongyloides stercoralis* larvae in BAL established the diagnosis and supports an infectious mechanism of alveolar injury. In endemic regions, this highlights the importance of including parasitic infections in the differential diagnosis of DAH, particularly in immunocompromised patients. From a diagnostic perspective, early bronchoscopy with BAL plays a central role in confirming DAH [14,20].

In this case, the absence of early clinical suspicion contributed to delayed diagnosis and late initiation of therapy. In immunocompromised patients presenting with DAH, particularly in endemic settings and with recent corticosteroid exposure, a high index of suspicion for strongyloidiasis is essential. In previously reported cases of strongyloidiasis in Colombia, diagnosis has often been established at advanced stages of disease, suggesting that delays in recognition may occur and could influence clinical outcomes [18].

Current evidence supports the implementation of screening strategies prior to immunosuppressive therapy, especially in individuals from endemic areas, given the risk of life-threatening hyperinfection [22,23]. When access to diagnostic tools is limited or results are delayed, serological testing should be prioritized, and empiric ivermectin therapy may be considered in high-risk patients to prevent disease progression [24]. This case illustrates how delayed recognition and lack of pre-immunosuppression screening can directly impact outcomes, reinforcing the need for proactive diagnostic and therapeutic strategies in endemic settings.

In this case, treatment was initiated at an advanced stage of disease, which likely limited its effectiveness. Although intensified or repeated ivermectin regimens have been described, randomized trials in uncomplicated strongyloidiasis have shown no clear advantage of multiple-dose strategies over standard dosing, with similar efficacy outcomes [25]. Similarly, alternative routes of administration, such as subcutaneous or rectal ivermectin, have been reported in selected cases with impaired absorption, but available data are limited to case reports and lack standardized protocols or proven benefit in mortality reduction [26]. Moreover, evidence in severe or disseminated disease remains limited to case reports and small series, without robust data demonstrating improved survival with higher or combined dosing approaches [27]. Given the persistently high mortality reported in hyperinfection syndrome, these findings suggest that delayed diagnosis and advanced disease stage may have a greater impact on outcomes than the specific ivermectin regimen used.

Albendazole (400 mg twice daily) remains a second-line option due to its lower efficacy compared with ivermectin and is generally reserved for situations in which ivermectin is unavailable [28].

In addition, reduction in or discontinuation of immunosuppressive therapy should be considered whenever clinically feasible, as ongoing corticosteroid exposure sustains the autoinfective cycle and reduces treatment effectiveness. Finally, bacterial translocation associated with larval migration increases the risk of Gram-negative bacteremia, requiring prompt antimicrobial therapy guided by microbiological findings [17,29].

In this case, the absence of pre-immunosuppression screening likely contributed to delayed diagnosis and poor outcome. Preventive strategies aimed at avoiding hyperinfection—particularly the administration of ivermectin prior to corticosteroid therapy—have been shown to be cost-effective and associated with reduced mortality in high-risk populations [30,31]. In endemic settings such as Colombia, screening for *Strongyloides stercoralis* should be performed before initiating corticosteroids or other immunosuppressive therapies. When serological testing is available, it is preferred; however, limited access and reduced sensitivity in immunosuppressed patients often restrict its utility. In these scenarios, empiric ivermectin represents a practical alternative [23,30]. Preventive strategies, including both screening and presumptive treatment, are cost-effective compared to no intervention, with empiric ivermectin often preferred in moderate-to-high prevalence settings due to its ability to prevent severe disease and deaths [32].

This case has several limitations. First, the lack of robust local epidemiological data limits a more precise contextualization of the burden of strongyloidiasis in Colombia. Second, despite the endemic setting, early clinical suspicion was limited, which may have contributed to delayed diagnosis. Finally, the interval between bronchoscopy and the availability of bronchoalveolar lavage cytology results may have delayed the initiation of targeted therapy, potentially influencing the clinical outcome.

## 4. Conclusions

This case highlights the need to systematically consider *Strongyloides stercoralis* hyperinfection in the differential diagnosis of DAH in endemic settings, especially among patients exposed to corticosteroids. Given the high associated mortality, early diagnosis and prompt treatment are critical. Preventive ivermectin administration prior to corticosteroid therapy should be increasingly incorporated into routine clinical practice, particularly in high-burden settings.

## Figures and Tables

**Table 1:** Laboratory findings at the time of respiratory deterioration (hospital day 25).

Parameter	Value	Reference Range
Leukocytes	31.12	4.0–10.0 × 10^3^/µL
Neutrophils	29.40	1.5–7.5 × 10^3^/µL
Lymphocytes	0.44	1.0–4.0 × 10^3^/µL
Monocytes	0.59	0.2–0.8 × 10^3^/µL
Eosinophils	0.44	0.0–0.5 × 10^3^/µL
Hemoglobin	7.0	13.0–17.0 g/dL
Hematocrit	21.3	40–50%
Mean corpuscular volume	97.3	80–100 fL
Platelets	101	150–400 × 10^3^/µL
Erythrocyte sedimentation rate	2	<20 mm/h
C-reactive protein	89.8	<5 mg/L
Creatinine	0.7	0.7–1.3 mg/dL
Blood urea nitrogen	23.6	7–20 mg/dL
Alkaline phosphatase	107	40–130 U/L
Alanine aminotransferase	73	<40 U/L
Aspartate aminotransferase	65	<40 U/L
Total bilirubin	1.1	0.3–1.2 mg/dL
Direct bilirubin	0.6	0.0–0.3 mg/dL
Indirect bilirubin	0.5	0.2–0.9 mg/dL

**Table 2:** Clinical timeline.

Timepoint	Clinical Events
2 months before admission	Progssrive lower limb pain with associated weakness
Admission (Day 0)	Neurological deficits; HIV serology negative; MRI revealed lumbosacral mass with spinal cord compression; initiation of intravenous dexamethasone (8 mg every 12 h)
Days 1–20	Biopsy confirmed metastatic urothelial carcinoma
Day 25	Acute respiratory deterioration with hypoxemia and shock; ICU admission; orotracheal intubation; vasopressor support; initiation of broad-spectrum antibiotics; chest imaging showing bilateral ground-glass opacities
Day 26	BAL showing DAH
Day 30	BAL cytology confirmed *Strongyloides stercoralis*; initiation of oral ivermectin (200 µg/kg once daily)
Day 31	Death due to rapid clinical deterioration

## Data Availability

The data supporting the findings of this case report are not publicly available due to patient privacy and ethical restrictions. Relevant anonymized data may be available from the corresponding author upon reasonable requestt.

## References

[1] Buonfrate D., Bisanzio D., Giorli G., Odermatt P., Fürst T., Greenaway C., French M., Reithinger R., Gobbi F., Montresor A. (2020). The Global Prevalence of *Strongyloides stercoralis* Infection. Pathogens.

[2] Buonfrate D., Mena M.A., Angheben A., Requena-Mendez A., Munõz J., Gobbi F., Albonico M., Gotuzzo E., Bisoffi Z., The COHEMI Project Study Group (2015). Prevalence of strongyloidiasis in Latin America: A systematic review of the literature. Epidemiol. Infect..

[3] Czeresnia J.M., Weiss L.M. (2022). *Strongyloides* *stercoralis*. Lung.

[4] Page W., Judd J.A., Bradbury R.S. (2018). The Unique Life Cycle of *Strongyloides stercoralis* and Implications for Public Health Action. Trop. Med. Infect. Dis..

[5] Albarqi M.M.Y., Stoltzfus J.D., Pilgrim A.A., Nolan T.J., Wang Z., Kliewer S.A., Mangelsdorf D.J., Lok J.B. (2016). Regulation of Life Cycle Checkpoints and Developmental Activation of Infective Larvae in *Strongyloides stercoralis* by Dafachronic Acid. PLoS Pathog..

[6] Stoltzfus J.D., Bart S.M., Lok J.B. (2014). cGMP and NHR signaling co-regulate expression of insulin-like peptides and developmental activation of infective larvae in *Strongyloides stercoralis*. PLoS Pathog..

[7] Herbert D.R., Stoltzfus J.D.C., Rossi H.L., Abraham D. (2022). Is Strongyloides stercoralis hyperinfection induced by glucocorticoids a result of both suppressed host immunity and altered parasite genetics?. Mol. Biochem. Parasitol..

[8] Jaleta T.G., Lok J.B. (2019). Advances in the Molecular and Cellular Biology of *Strongyloides* spp. Curr. Trop. Med. Rep..

[9] Kassalik M., Mönkemüller K. (2011). *Strongyloides stercoralis* Hyperinfection Syndrome and Disseminated Disease. Gastroenterol. Hepatol..

[10] Kim E.J. (2018). Acute Respiratory Distress Syndrome With Alveolar Hemorrhage due to Strongyloidiasis Hyperinfection in an Older Patient. Ann. Geriatr. Med. Res..

[11] Lam C.S., Tong M.K.H., Chan K.M., Siu Y.P. (2006). Disseminated strongyloidiasis: A retrospective study of clinical course and outcome. Eur. J. Clin. Microbiol. Infect. Dis..

[12] Steinhaus D.A., Gainor J.F., Vernovsky I., Winsett J., Beer D.J. (2012). Survival in a case of diffuse alveolar hemorrhage due to *Strongyloides stercoralis* hyperinfection. Respir. Med. Case Rep..

[13] Qu T.T., Yang Q., Yu M.H., Wang J. (2016). A Fatal *Strongyloides Stercoralis* Hyperinfection Syndrome in a Patient with Chronic kidney Disease: A Case Report and Literature Review. Medicine.

[14] El-Sameed Y.A., Beejay N., Al Maashari R. (2015). Diffuse alveolar haemorrhage and severe hypoxemia from *Strongyloides stercoralis* hyperinfection syndrome. Clin. Respir. J..

[15] Dharmalingam A., Ayub I.I., Subramanian P.S.M., Bhaskar M.E. (2026). Strongyloidiasis hyperinfection presenting as diffuse alveolar hemorrhage in a patient with systemic lupus erythematosus. Can. J. Respir. Crit. Care Sleep Med..

[16] Gonzalez-Ibarra F., Chevli P., Schachter L., Kaur M., Eivaz-Mohammadi S., Tashtoush B., Matta J., Syed A.K., Marian V. (2014). Strongyloidiasis and Diffuse Alveolar Hemorrhage in a Patient with Systemic Lupus Erythematosus. Case Rep. Med..

[17] Krolewiecki A., Nutman T.B. (2019). Strongyloidiasis: A Neglected Tropical Disease. Infect. Dis. Clin. N. Am..

[18] Vinueza D., Collazos-Torres L.A., Vallejo-Serna R.A., Gómez-Gil B.S., Quintero-Romero J.M., Muñoz-Lombo J.P. (2026). *Strongyloides stercoralis*: From chronic silent infection to fulminant catastrophe. Int. J. Infect. Dis..

[19] Rivera A., Patiño M., Ocampo J.M., Suárez J., López G., Salazar W. (2021). Strongyloides stercolaris hyperinfection in a young patient with HTLV-1 infection and ulcerative colitis. Rev. Colomb. Gastroenterol..

[20] Krause M.L., Cartin-Ceba R., Specks U., Peikert T. (2012). Update on Diffuse Alveolar Hemorrhage and Pulmonary Vasculitis. Immunol. Allergy Clin. N. Am..

[21] Park M.S. (2013). Diffuse Alveolar Hemorrhage. Tuberc. Respir. Dis..

[22] de Souza J.N., Inês E.D.J., Santiago M., Teixeira M.C.A., Soares N.M. (2016). *Strongyloides stercoralis* infection in patients with systemic lupus erythematosus: Diagnosis and prevention of severe strongyloidiasis. Int. J. Rheum. Dis..

[23] Mejia R., Nutman T.B. (2012). Screening, prevention, and treatment for hyperinfection syndrome and disseminated infections caused by *Strongyloides stercoralis*. Curr. Opin. Infect. Dis..

[24] Lucas-Dato A., Hernández-Rabadán M.D., Arroyo P.L.B., Llenas-García J. (2025). Hyperinfection by *Strongyloides stercoralis*: Series of Cases in a Regional Hospital in Southern Spain. Microbiol. Res..

[25] Buonfrate D., Salas-Coronas J., Muñoz J., Maruri B.T., Rodari P., Castelli F., Zammarchi L., Bianchi L., Gobbi F., Cabezas-Fernández T. (2019). Multiple-dose versus single-dose ivermectin for *Strongyloides stercoralis* infection (Strong Treat 1 to 4): A multicentre, open-label, phase 3, randomised controlled superiority trial. Lancet Infect. Dis..

[26] Bogoch I.I., Khan K., Abrams H., Nott C., Leung E., Fleckenstein L., Keystone J.S. (2015). Failure of Ivermectin per Rectum to Achieve Clinically Meaningful Serum Levels in Two Cases of Strongyloides Hyperinfection. Am. J. Trop. Med. Hyg..

[27] Rojas O.C., Montoya A.M., Villanueva-Lozano H., Carrion-Alvarez D. (2023). Severe strongyloidiasis: A systematic review and meta-analysis of 339 cases. Trans. R. Soc. Trop. Med. Hyg..

[28] Henriquez-Camacho C., Gotuzzo E., Echevarria J., Clinton White A., Terashima A., Samalvides F., Perez-Molina J.A., Plana M.N. (2016). Ivermectin versus albendazole or thiabendazole for *Strongyloides stercoralis* infection. Cochrane Database Syst. Rev..

[29] Bisoffi Z., Buonfrate D., Montresor A., Requena-Méndez A., Muñoz J., Krolewiecki A.J., Gotuzzo E., Mena M.A., Chiodini P.L., Anselmi M. (2013). *Strongyloides stercoralis*: A Plea for Action. PLoS Negl. Trop. Dis..

[30] Yongbantom A., Sribenjalux W., Manomaiwong N., Meesing A. (2023). Efficacy of Oral Ivermectin as Empirical Prophylaxis for Strongyloidiasis in Patients Treated with High-Dose Corticosteroids: A Retrospective Cohort Study. Am. J. Trop. Med. Hyg..

[31] Stauffer W.M., Alpern J.D., Walker P.F. (2020). COVID-19 and Dexamethasone: A Potential Strategy to Avoid Steroid-Related Strongyloides Hyperinfection. JAMA.

[32] Joo H., Maskery B.A., Alpern J.D., Weinberg M., Stauffer W.M. (2024). Cost-effectiveness of treatment strategies for populations from strongyloidiasis high-risk areas globally who will initiate corticosteroid treatment in the United States. J. Travel. Med..

